# Preventive Effects of Catechins on Cardiovascular Disease

**DOI:** 10.3390/molecules21121759

**Published:** 2016-12-21

**Authors:** Xiao-Qiang Chen, Ting Hu, Yu Han, Wei Huang, Hai-Bo Yuan, Yun-Tian Zhang, Yu Du, Yong-Wen Jiang

**Affiliations:** 1 College of Bioengineering and Food, Hubei University of Technology, Wuhan 430068, China; huting1988@163.com (T.H.); wyyx2016277@163.com (Y.H.); jinan1616@163.com (Y.-T.Z.); ddddy11@icloud.com (Y.D.); 2 Hubei Institute of Food and Drug Control, Wuhan 430075, China; yifan_tj@163.com; 3 Tea Research Institute, China Academy of Agricultural Sciences, Hangzhou 310008, China; 192168092@tricaas.com

**Keywords:** catechins, cardiovascular disease, health care

## Abstract

Catechins are polyphenolic phytochemicals with many important physiological activities that play a multifaceted health care function in the human body, especially in the prevention of cardiovascular disease. In this paper, various experimental and clinical studies have revealed the role of catechins in the prevention and treatment of cardiovascular disorders, and we review the preventive effects of catechins on cardiovascular disease from the following aspects: Regulating lipid metabolism, regulating blood lipid metabolism, vascular endothelial protection, and reducing blood pressure.

## 1. Introduction

Catechins are polyphenolic phytochemicals that are widely distributed in food and medicinal plants, especially in all kinds of tea, and endow tea with various health benefits [1,2,3]. A growing number of studies have linked intake of catechins-rich foods with the prevention of human chronic diseases, and laboratory studies have demonstrated the potential preventive activity of catechins in laboratory models of cardiovascular disease [4]. In general, cardiovascular diseases and cerebrovascular diseases are collectively called cardiovascular disease, which are caused by hyperlipidemia, high blood viscosity, atherosclerosis, hypertension, and so on, which lead to ischemic or hemorrhagic disease [5,6]. Nowadays, cardiovascular disease has become a common disease. Irregular life and unhealthy diets cause more people to suffer from cardiovascular diseases, such as obesity, fat deposits, and so on [7]. Tea contains catechins, caffeine, and many kinds of beneficial active materials, which play an important role in human health [8,9,10]. In particular, catechins have a significant effect on the prevention of cardiovascular disease [11,12,13]. Many clinical and epidemiological studies have examined the actions of catechins in terms of anti-carcinogenic [14], anti-tumorigenic [15], anti-mutagenic [16], anti-diabetic [17], and anti-obesity effects [18]. This paper has reviewed the preventive effects of catechins on cardiovascular disease, which were published in the past few decades.

## 2. Catechins

**Figure 1:**
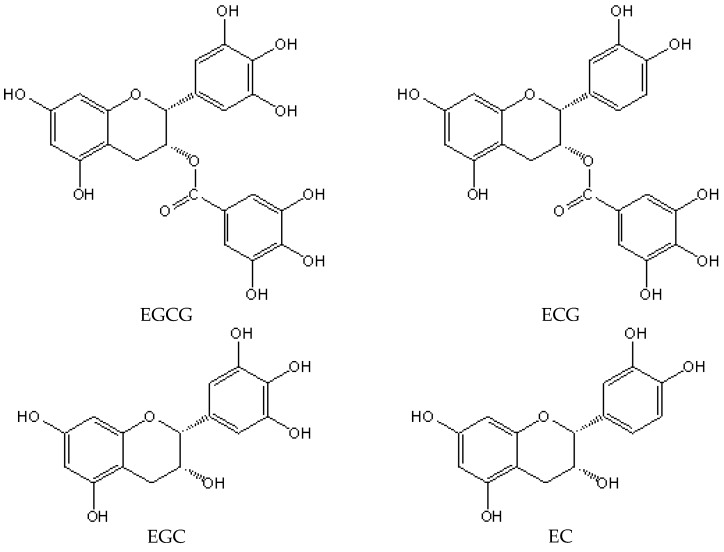
The structures of four kinds of catechins: epicatechin (EC), epigallocatechin (EGC), epicatechin gallate (ECG), and epigallocatechin gallate (EGCG) [25].

Catechins are the generic terms of flavanols, as well as the most important substance of tea polyphenol, which is mainly divided into four kinds of compounds (Figure 1): epicatechin (EC), epigallocatechin (EGC), epicatechin gallate (ECG), and epigallocatechin gallate (EGCG) [19]. EGCG is the most abundant kind of tea catechin, and is thought to be responsible for the majority of the biological activities that are detected. Because they possess ortho dihydroxy or ortho trihydroxy structure, catechins could form half quinone free radicals when they act on free radicals, leading to harmful oxygen free radical inactivation [20]. Chelate reaction of catechins with oxidizing metal ions could inhibit the oxidation activity of metal ions due to its ortho hydroxyl phenol structure [21,22]. The versatility of catechins and their active metabolites as potential therapeutic interventions are due to the diverse actions being performed at different sites. Because of the special structure, catechins are endowed with strong antioxidant activity and unique effects on the prevention of cardiovascular disease [23,24].

## 3. Catechins Regulate Lipid Metabolism

Obesity is an antecedent of various diseases. It easily causes myocardial infarction, cerebral thrombosis, coronary heart disease, and other cardiovascular diseases [24,25,26,27]. Obesity can increase the risk to the human body by reducing the total vascular resistance below normal limits and increase the burden on the heart [28,29]. According to a contrast test of obese and normal children in Japan for 24 weeks, it was found that the intake of catechins at a rate of 576 mg per day can significantly decrease body fat. Catechins could regulate the RNA and protein expression of fatty acid metabolism enzymes in the liver to affect the activity of metabolic enzymes, which can improve the oxidation of fatty acids and the inhibition of fatty acid synthesis, so as to reduce the lipid levels in the blood and liver, as well as decrease body fat deposition, thus reducing early onset of cardiovascular disease in children [30]. For the regulation mechanism of catechins on obesity, Dulloo et al. reported that catechins could control lipid accumulation by adjusting the sympathetic nervous system (SNS) or interfering with the SNS and neurotransmitter norepinephrine lipids, which significantly improved the body fat ratio and reduced the risk of cardiovascular disease caused by obesity [31].

## 4. Catechins Regulate Blood Lipid Metabolism

Lipid metabolic disorders are the pathologic antecedent of atherosclerosis (AS), which is the main cause of coronary heart disease, cerebral infarction, and peripheral vascular disease [32,33,34]. Hyperlipidemia plays a promoting role in the risk of AS, and the increased lipid peroxide (LPO) in serum may lead to platelet aggregation in vivo, with the result that it will cause AS in the damaged body [35,36]. Ruidavets et al. reported that catechins could enhance the activities of antioxidant enzymes by scavenging excess free radicals and improving the vitality of intracellular catalase and superoxide dismutase to inhibit oxidative damage, reducing lipid peroxidation to protect the body, based on participants’ blood samples [37]. In addition, EGCG was shown to promote the expression of p53, p21, and NF-κB, thus inducing the apoptosis of vascular smooth muscle cells so as to inhibit the development of atherosclerosis [38]. In addition, catechins can reduce the accumulation of cholesterol and its oxidation products in artery walls when it is combined with free radicals in vivo, thus improving blood circulation in order to play a part in the prevention of AS [39]. Studies have found that different kinds of catechins may improve the levels of serum triglyceride, total cholesterol, low-density lipoprotein cholesterol, and apolipoprotein B, thereby reducing the blood fat deposition [40]. Moreover, catechins can catalyze the activities of superoxide dismutase in serum and liver, as well as exert its oxidation resistance through the formation of half quinone radicals [41,42]. Further, it was found that EGCG could inhibit the oxidation of low density lipoprotein (LDL) induced by Cu^2+^ and increase its anti-oxidation in vivo, thus reducing the risk of cardiovascular disease [43].

## 5. Protection of Vascular Endothelium from Catechins 

The vascular endothelium is a simple monolayer of inner blood vessels that separates blood and peripheral tissues, and it maintains homeostasis by regulating vascular tone [44]. Vascular endothelium can maintain blood vessel balance and protect endothelial tissue from lipid deposition, and can cause vascular endothelium thickening and hardening, which result in narrow blood vessels, occlusion, and myocardial blood supply insufficiency, which can lead to hypertension and other kinds of cardiovascular disease [45,46,47,48].

**Figure 2:**
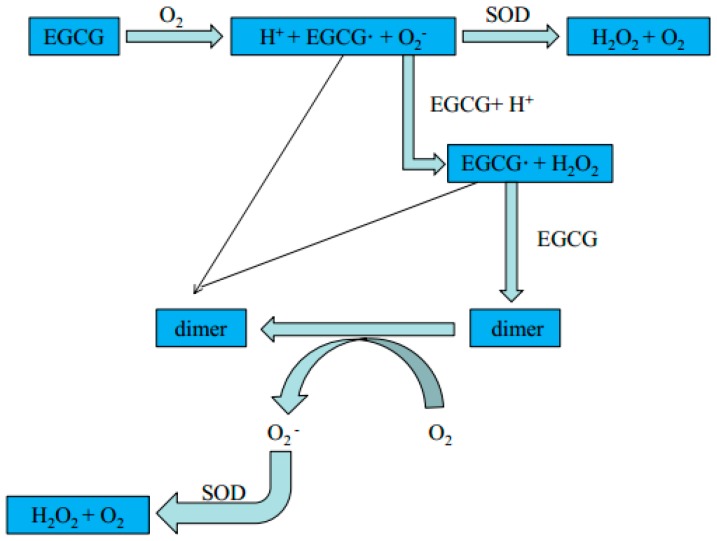
Mechanism of EGCG auto-oxidation and dimerization.

Catechins play an important role in the improvement of the vascular endothelial environment and in the prevention of dysfunction, which mainly works through the following ways so as to eliminate excessive active free radicals in order to prevent the oxidation of lipids in the human body [49,50]. The active sites of EGCG chelate with metal ions to form an inactivated complex that could reduce the catalytic effects of metal ions in the oxidation reaction and effectively remove surplus active free radicals from the human body, thus reducing the damage to the vascular endothelium and decrease the possibility of thrombosis (Figure 2) [51]. High-speed electrons are transferred by EGCG at the site of reactive oxygen species (ROS)-induced free radical so as to reduce the oxidation reaction. Meanwhile, EGCG is easily oxidized, and can form a quinone structure on the B-ring or D-ring, and then it will form stable half quinone free radicals, thereby reducing the oxidation reaction [52]. Nitric oxide (NO)—an endothelium-derived relaxing factor—is generated during the conversion of l-arginine to l-citrulline by endothelial NO synthase in the presence of various substrates and co-factors [53]. NO is a fundamental determinant for maintaining vascular function. Catechins can stabilize NO when blood vessels are damaged, and 10 μg/mL of EC could cause the phosphorylation of nitric oxide synthase serine residues 633 and 1177, as well as the dephosphorylation of threonine residues 495, which can strengthen the activity of eNOS and increase the release of NO, and then delay the signals from hemodynamic changes accepted by endothelial cells in the body, which avoid endothelial cells from causing a strong stress response in order to reduce the formation of blood clots [54,55]. Improvements in vascular endothelial dysfunction might potentially contribute to the beneficial effects of tea catechins in the treatment of patients with hypertension.

## 6. Catechins Regulate Hypertension

Hypertension is one of the most important factors that induce cardiovascular diseases. It increases the burden on the heart and blood vessel wall pressure when blood pressure is higher than the normal range, which can damage blood vessels and very easily induce cardiovascular diseases [56,57]. Epidemiological studies, clinical intervention trials, and animal experiments have confirmed that catechins have very strong antihypertensive activity [58,59,60]. Catechins can regulate nicotinamide adenine dinucleotide phosphate (NADPH) oxidase gene subtype and promote the expression of heme oxygenase so as to maintain blood pressure balance in the body [61]. Studies have found that catechins can inhibit RNA expression of IL-6 and MMP-9 in the serum of patients suffering from hypertension, which benefits a lower blood pressure [62]. Additionally, it is more effective for EGCG to inhibit the activity of calcium-activated chloride channels than other kinds of catechins, which could improve calcium signals in the regulation of phosphorylation levels of inositol triphosphate, calmodulin-dependent protein kinase II, and calmodulin antibodies; thus, it possesses strong antihypertensive activity [63,64].

## 7. Prospects

The research into the internal substances contained in tea is a new direction that has been rapidly developed in recent years, especially in the study of catechins, which have made an important breakthrough and been given a great deal of attention by the government of developed countries and by the scientific community. Nowadays, hypotensive and antiphlogistic drugs made from catechins have been successfully and preliminarily developed. There are still many problems that need to be solved in the direction of catechins, which will be applied in the prevention of cardiovascular disease. However, this application is likely to produce a great social and economic benefit once research yields breakthroughs. Moreover, it will have a profound impact on modernization and internationalization.
